# Mucin 21 confers resistance to apoptosis in an *O*-glycosylation-dependent manner

**DOI:** 10.1038/s41420-022-01006-4

**Published:** 2022-04-11

**Authors:** Yuan Tian, Kaori Denda-Nagai, Tatsuya Tsukui, Katrin B. Ishii-Schrade, Kyoko Okada, Yoshihiro Nishizono, Kosuke Matsuzaki, Margarete Hafley, Robert S. Bresalier, Tatsuro Irimura

**Affiliations:** 1 Laboratory of Cancer Biology and Molecular Immunology, Graduate School of Pharmaceutical Sciences, The University of Tokyo, 7-3-1 Hongo, Bunkyo-ku, Tokyo, 113-0033 Japan; 2 Division of Glycobiologics, Intractable Disease Research Center, Juntendo University, 2-1-1 Hongo, Bunkyo-ku, Tokyo, 113-8421 Japan; 3 Department of Gastroenterology, Hepatology and Nutrition, The University of Texas MD Anderson Cancer Center, 1515 Holcombe Boulevard Unit 1466, Houston, TX 77030 USA

**Keywords:** Glycobiology, Glycobiology

## Abstract

Highly glycosylated mucins protect epithelial surfaces from external insults and are related to malignant behaviors of carcinoma cells. However, the importance of carbohydrate chains on mucins in the process of cellular protection is not fully understood. Here, we investigated the effect of human mucin-21 (MUC21) expression on the susceptibility to apoptosis. MUC21 transfection into HEK293 cells decreased the number of apoptotic cells in culture media containing etoposide or after ultraviolet light irradiation. We used Chinese hamster ovary (CHO) cell variants to investigate the importance of MUC21 glycosylation in the resistance to apoptosis. When MUC21 was expressed in CHO-K1 cells, it was glycosylated with sialyl T-antigen and the cells showed resistance to etoposide-induced apoptosis. MUC21 transfection into Lec2 cells, a variant of CHO cells lacking sialylation of glycans, revealed that the presence of nonsialylated T-antigen also renders cells resistant to etoposide-induced apoptosis. MUC21 was transfected into ldlD cells and the glycosylation was manipulated by supplementation to the medium. Nonsupplemented cells and cells supplemented with *N*-acetylgalactosamine showed no resistance to etoposide-induced apoptosis. In contrast, these cells supplemented with *N*-acetylgalactosamine plus galactose expressed sialyl T-antigen and exhibited resistance to etoposide-induced apoptosis. Finally, galectin-3 knockdown in MUC21 transfectants of HEK293 cells did not significantly affect MUC21-dependent induction of apoptosis resistance. The results suggest that T-antigen with or without sialic acid is essential to the antiapoptotic effect of MUC21.

## Abstract (web summary)

Mucin 21 (MUC21) is a large glycoprotein that protects squamous epithelia. Glycan changes in mucins occur in cancer cells and are thought to contribute to malignant progression. We report glycoform-dependent antiapoptotic effects of MUC21. Various MUC21 glycoforms were expressed in HEK293 and CHO cells. Apoptosis was induced using etoposide or UV exposure. MUC21 with glycans terminated with galactose/sialic acid inhibited apoptosis; MUC21 with no glycans or *N*-acetylgalactoseamine did not.

## Graphical abstract

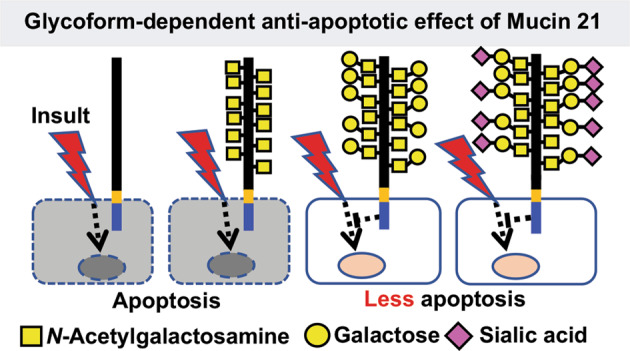

## Introduction

Mucins are highly *O*-glycosylated proteins thought to protect epithelial surfaces from physical, chemical, and biological insults. Attention to mucins has been paid in the fields of cancer diagnosis and cancer therapy, where some mucins such as mucin 1 (MUC1) and MUC16 have widely been used as serum diagnostic markers [1–3]. Accumulating evidence shows that cells become resistant to apoptotic processes when MUC1, MUC4, or MUC13 are expressed, implicating mucin expression in the malignant behaviors of carcinoma cells such as resistance to therapeutic modalities [4–7].

Mucin 21 (MUC21) is a unique transmembrane-type mucin, found in an endeavor to identify mouse epiglycanin, a mammary carcinoma-associated mucin. Strong associations between the malignant behavior of mouse mammary carcinoma TA3-Ha cells and the presence of epiglycanin were reported [8–10]. We identified a novel transmembrane mucin as the molecular entity of epiglycanin, found the human counterpart and named it as MUC21 [11]. Although epiglycanin was reported to have immune-suppressive functions [12], we have not thus far been able to reproduce similar results with the cloned gene. MUC21 is a distinct mucin both in its structure and distribution. Its tandem repeats consist of repetitive but not identical 15 amino acids, seven or eight of which are serine or threonine. Public databases report a unique tissue localization, clearly indicating that it is a product of squamous epithelia, where epithelial cells are typically exposed to high levels of physical stress. Our previous investigations revealed that mouse Muc21 has a strong anti-adhesive function when expressed on a variety of cells [13]. Further, we recently reported a characteristically high MUC21 protein expression in cancer cells of patients with incohesive-type lung adenocarcinoma as opposed to other, cohesive-type lung adenocarcinomas [14, 15]. These observations led us to speculate that MUC21 has an antiapoptotic activity similar to MUC1, MUC4, and MUC13.

The hallmark of mucins is their high content of *O*-glycans and tandem-repeat structures with high proportions of serine and threonine, the *O*-glycosylation sites. However, investigations on the mechanism of resistance to apoptosis by the expression of mucins have been focused on cellular signals mediated by their cytoplasmic tails. For example, overexpression of MUC1 confers sustained induction of the IKKβ–NF-κB p65 pathway [16]. Involvement of mucins in tumor resistance to chemotherapeutic drugs is extensively reviewed by Jonckheere and coworkers [17]. Similarly, Reynolds and coworkers recently conducted a systematic literature review on the apoptosis-blocking function of mucin glycoproteins and identified 90 studies which show evidence that MUC1, MUC2, MUC4, MUC5AC, MUC5B, MUC13, and MUC16 are involved in apoptosis resistance or cancer drug resistance in epithelial cancer cells [18]. However, none of these studies investigated the involvement of mucin glycosylation in the mechanism of apoptosis resistance, which clearly illustrates how little is known about the role of the tandem-repeat domain and its glycosylation in the resistance to apoptosis that is conferred by the expression of membrane-type and secreted-type mucins. It has been shown that *O*-glycosylation of the MUC1 extracellular domain contributes to anoikis resistance in epithelial cancer cells [19, 20]. However, the authors of these studies did not determine if a specific glycoform of MUC1 was responsible for this phenomenon.

Here, we report that expression of MUC21 by transfection makes epithelial cells resistant to apoptosis. Both the extracellular and the cytoplasmic domain are necessary for this effect. Furthermore, for this effect to occur, MUC21’s *O*-glycans need to be extended to contain galactose (Gal) residues that may or may not be sialylated. In addition, we show that MUC21-dependent apoptosis resistance is not altered in the absence of galectin-3, suggesting that another galectin or carbohydrate-binding molecule is mediating the effect. This is the first report to show that the antiapoptotic effect of a membrane-type mucin depends on the extension of its *O*-linked carbohydrate chains.

## Results

### Expression of MUC21 makes HEK293 cells resistant to apoptosis induced by etoposide

**Fig. 1:**
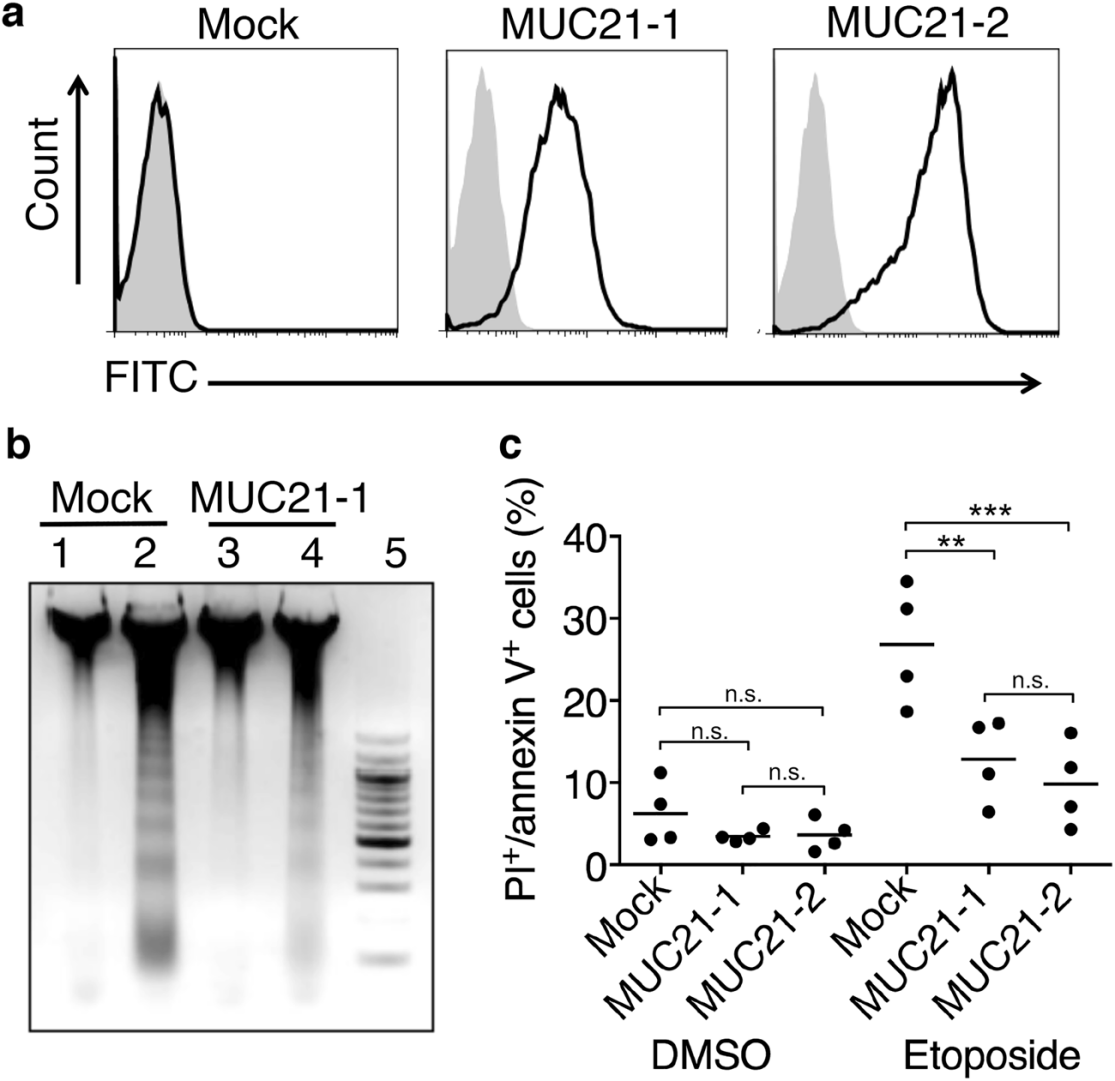
MUC21 expression renders cells resistant to apoptosis. **a** Flow cytometric profiles of two clonal populations (MUC21-1 and MUC21-2) and mock transfectant of HEK293 cells transfected with full-length human MUC21. The cells were stained with anti-MUC21 monoclonal antibody heM21C followed by FITC-labeled goat anti-mouse IgG (solid line) and by control mouse IgG (shaded area). **b** Results of agarose gel electrophoresis of DNA extracted from HEK293 cells untreated (lanes 1 and 3) or treated (lanes 2 and 4) with 100 µM etoposide for 24 h. The cell clones for Mock and MUC21 are the same with the clones shown in (**a**). Lane 5 shows the molecular weight marker ladder. (**c**) Percentage of apoptotic cells among mock-transfectant cells and two clones transfected with full-length MUC21 (MUC21-1 and MUC21-2) as determined by flow cytometric analysis 48 h after incubation with DMSO or 100 µM etoposide. Four independent experiments are shown. The mean is indicated as a horizontal line. Two-way ANOVA with Tukey’s multiple-comparison test. **p < 0.01, ***p < 0.001, n.s. not significant.

Clonal populations were obtained from HEK293 cells transfected with full-length MUC21 cDNA and the resultant expression of MUC21 was confirmed by flow cytometry using monoclonal antibody (mAb) heM21C (Fig. 1a). Mock-transfected and MUC21-transfected cells were treated with 100 μM etoposide for 48 h. DNA extracted from untreated and etoposide-treated cells was run on an agarose gel to visualize DNA degradation resulting from apoptosis. Figure 1b shows that DNA from MUC21-transfected cells was less degraded than DNA from mock-transfected cells. Etoposide-treated cells were also subjected to flow cytometric analysis with propidium iodide (PI)/annexin V. Figure 1c shows that MUC21-transfected cells contained a lower percentage of apoptotic cells, indicating that MUC21 expression renders cells resistant to etoposide-induced apoptosis.

### Both tandem-repeat and cytoplasmic domains are necessary for the antiapoptotic effect of MUC21

**Fig. 2:**
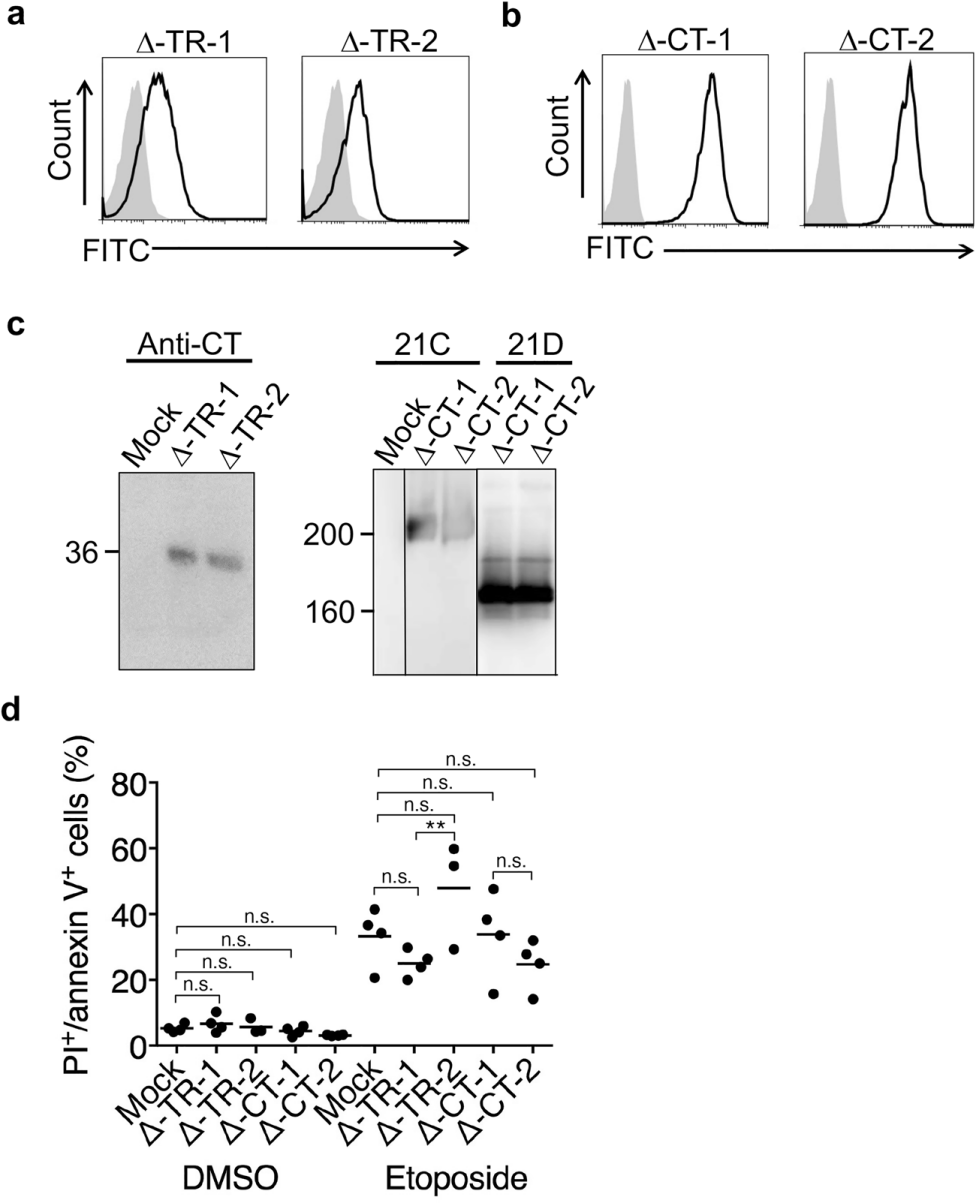
The extracellular domain and the cytoplasmic tail are essential in the antiapoptotic effect of MUC21. **a** Flow cytometric analysis of two clonal populations of HEK293 transfectants with MUC21 lacking the tandem- repeat domain with rabbit anti-CT polyclonal antibody (Anti-CT) after permeabilization. **b** Flow cytometric analysis of two clonal populations of HEK293 transfectants with MUC21 lacking the cytoplasmic tail domain with a 1:1 mixture of mAbs heM21C (21C) and heM21D (21D). The solid line represents antibody binding and the shaded area represents isotope control antibody binding. **c** Western blotting analysis of electrophoretically separated lysates of mock-transfected cells and two clones of transfectants with MUC21 lacking the tandem- repeat domain (Δ-TR-1 and Δ-TR-2, left panel), and mock-transfected cells and two clones of transfectants with MUC21 lacking the cytoplasmic tail (Δ-CT-1 and Δ-CT-2, right panel). **d** Percentage of mock transfectants, two clones of transfectants with MUC21 lacking the tandem-repeat domain (Δ-TR-1 and Δ-TR-2) and two clones of transfectants with MUC21 lacking the cytoplasmic tail (Δ-CT-1 and Δ-CT-2) undergoing apoptosis after treatment with 100 µM etoposide for 48 h. Four independent experiments are shown. The mean is indicated as a horizontal line. Two-way ANOVA with Tukey’s multiple-comparison test. **p < 0.01, n.s. not significant.

**Fig. 3:**
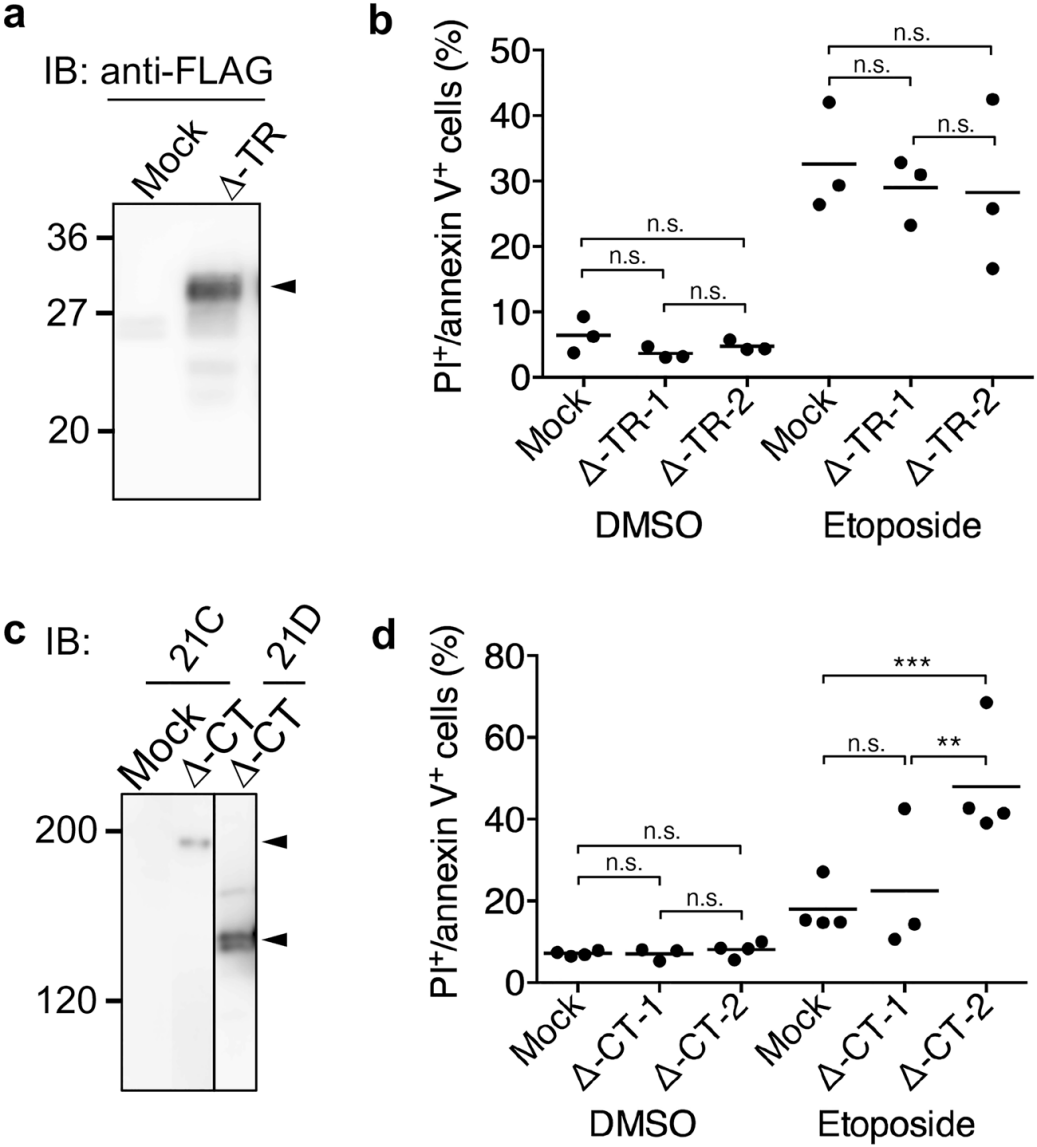
Truncated MUC21 shows no antiapoptotic effects when expressed by CHO-K1 cells. **a** Electrophoretic profiles of FLAG-tagged tandem-repeat-deficient MUC21 (Δ-TR) expressed by CHO-K1 cells. Lysates of mock transfectants and cells transfected with MUC21-Δ-TR were electrophoretically separated, blotted, and stained with biotinylated rabbit anti-FLAG antibody followed by streptavidine-labeled HRP. The arrow head indicates the band corresponding to Δ-TR-MUC21. **b** Percentage of apoptotic cells among two clones of CHO-K1 cells transfected with MUC21 deficient in the tandem-repeat domain (Δ-TR-1 and Δ-TR-2) or mock transfectant after treatment with 250 μM etoposide for 48 h as determined by PI staining and binding of annexin V. Three independent experiments are shown. **c** Electrophoretic profiles of FLAG-tagged cytosolic tail-deficient MUC21 (Δ-CT) expressed by CHO-K1 cells. Lysates of mock transfectants and cells transfected with Δ-CT were electrophoretically separated, blotted, and stained with biotinylated mAb heM21C (left panel) or mAb hM21D (right panel) followed by HRP-conjugated rabbit anti-mouse IgG. The arrowheads tentatively indicate fully glycosylated Δ-CT-MUC21 (top) and nonglycosylated Δ-CT-MUC21 (bottom). **d** Percentage of apoptotic CHO-K1 cells transfected with Δ-CT or mock transfectant after treatment with 250 μM etoposide for 48 h as determined by PI staining and binding of annexin V. Three and four independent experiments are shown. The mean is indicated as a horizontal line. Two-way ANOVA with Tukey’s multiple-comparison test. **p < 0.01, ***p < 0.001, n.s. not significant.

To investigate which part of MUC21 is responsible for inducing the antiapoptotic effect, MUC21 transfectant lacking the transmembrane domain (Δ-TR-MUC21) and another MUC21 transfectant lacking the cytoplasmic tail (Δ-CT-MUC21) were prepared from HEK293 cells. Successful expression of Δ-TR-MUC21 in two clones was confirmed by flow cytometry using a polyclonal rabbit anti-MUC21CT antibody [11] (Fig. 2a). Similarly, expression of Δ-CT-MUC21 in two clones was confirmed with a 1:1 mixture of mAb heM21C, specific for MUC21 with Tn (serine/threonine-linked GalNAc), T (serine/threonine-linked Gal(β1–3)GalNAc), or sialyl T (serine/threonine-linked Neu5Ac Gal(β1–3)GalNAc), but not the unmodified core polypeptide of MUC21, and mAb heM21D, specific for the unmodified core polypeptide of MUC21 and MUC21 attached with GalNAc [21]. Thus, this antibody mixture recognizes a wide variety of MUC21 glycoforms (Fig. 2b). Polyclonal anti-CT antibody was then used in Western blotting to confirm the expression of Δ-TR-MUC21, which resulted in a band with a migration position corresponding to an apparent molecular weight of 36 kDa (Fig. 2c, left panel). Δ-TR-MUC21 was constructed to contain the cytoplasmic domain, the transmembrane domain, and a neck domain, but is devoid of tandem repeats. The neck domain harbors several potential *O*-glycosylation sites, which might explain why the observed apparent molecular size was slightly higher than that based on amino acid-size calculation. Similarly, mAb heM21C and mAb heM21D were each used in Western blotting to confirm the expression of Δ-CT-MUC21. mAb heM21C recognized a band with a migration position corresponding to an apparent molecular weight around and above 200 kDa, probably representing fully glycosylated MUC21, while mAb heM21D showed a strong band at a migration position corresponding to an apparent molecular weight of 160 kDa, likely reflecting the presence of nonglycosylated MUC21 (Fig. 2c, right panel). Mock transfectant, full-length MUC21 transfectant, Δ-TR-MUC21 transfectant, and Δ-CT-MUC21 transfectant were treated with 100 μM etoposide for 48 h and analyzed by flow cytometry using PI/annexin V. The results revealed that expression of Δ-TR-MUC21 or Δ-CT-MUC21 alone could not render the cells resistant to apoptosis (Fig. 2d), while full-length MUC21 transfectant (as shown earlier in Fig. 1c) could. To investigate if these findings are reproducible in another cell type, we treated CHO-K1 cells transfected with Δ-TR-MUC21 (Fig. 3a) or Δ-CT-MUC21 (Fig. 3c) with 100 μM etoposide for 48 h and analyzed the cells by flow cytometry using PI/annexin V. Similar to HEK293 cells, CHO-K1 cells transfected with truncated versions of MUC21 did not show any apoptosis resistance (Fig. 3b, d).

### Anti-cell death effect of full-length MUC21 was observed when cell death was induced in HEK293 cells by ultraviolet irradiation (UVC)

**Fig. 4:**
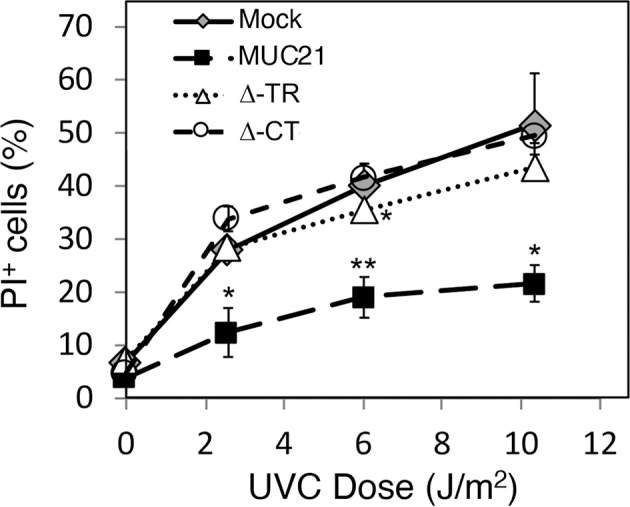
Anti-cell death effect of MUC21 was observed when cell death was induced in HEK293–MUC21 transfectants by ultraviolet irradiation. Dose–response curve of the effect of ultraviolet irradiation (UVC) on the percentage of propidium iodide-positive (PI+) cells in mock transfectants, MUC21 full-length transfectant (MUC21), transfectants with MUC21 lacking the tandem-repeat domain (Δ-TR), and transfectants with MUC21 lacking the cytosolic domain (Δ-CT). Means (SD) of three independent experiments are shown. Two-way ANOVA with Dunnett’s multiple-comparison test. *p < 0.05, **p < 0.01.

As another way to induce cell death, we utilized UVC irradiation emitted by a germicidal lamp inside the cell culture bench. Mock-transfectant and a panel of MUC21-transfectant cells were irradiated with varying doses of UVC light, after which dead cells were measured by flow cytometry using PI staining. In agreement with the results from the etoposide-induced apoptosis, expression of full-length MUC21 but not Δ-TR-MUC21 or Δ-CT-MUC21 alone could render the cells resistant to undergoing cell death (Fig. 4).

### CHO-K1 cells become resistant to etoposide when full-length MUC21 is expressed

**Fig. 5:**
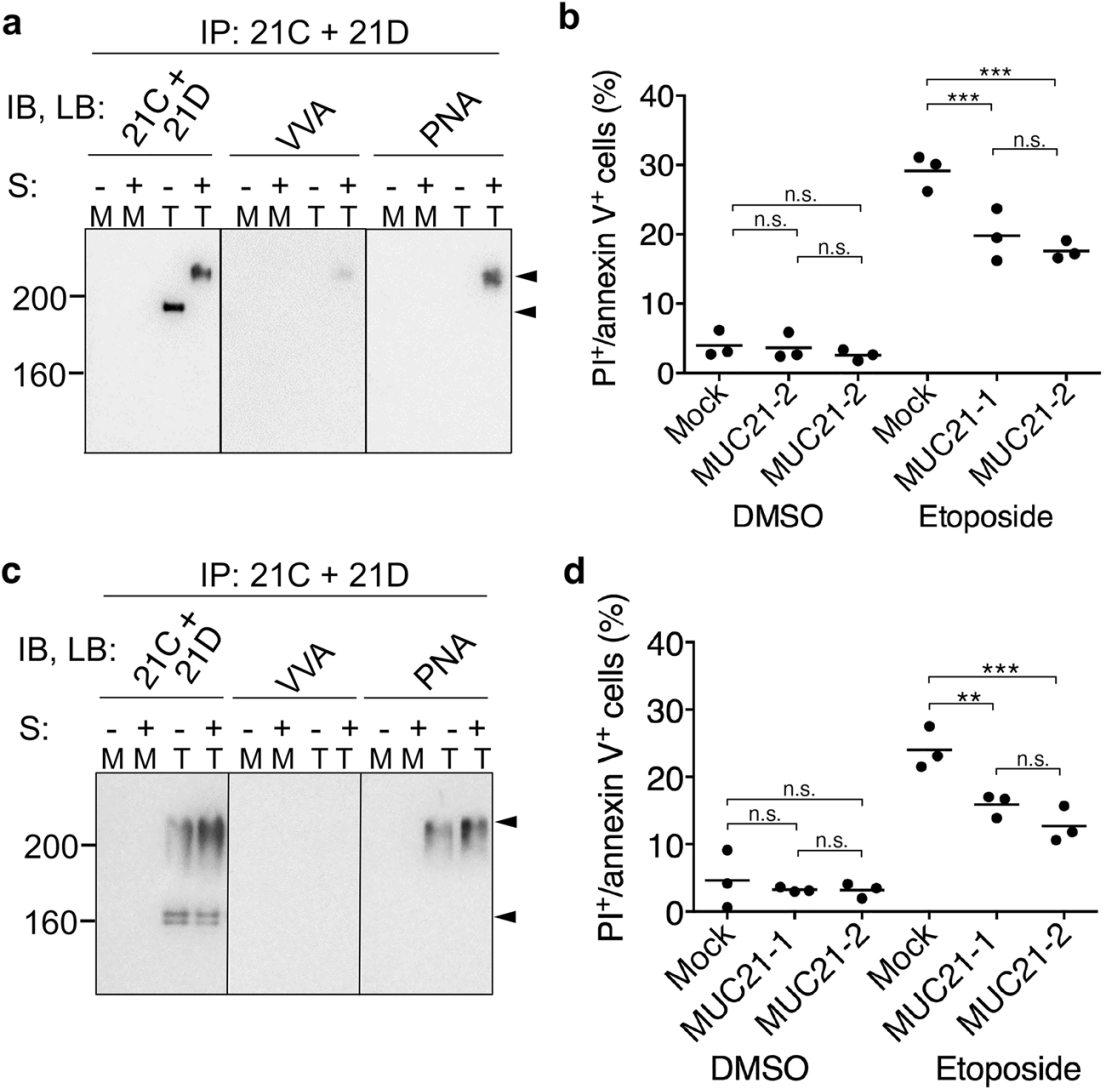
Glycosylation and antiapoptotic effects of MUC21 expressed on CHO-K1 cells and CHO-Lec2 cells. **a** Electrophoretic and lectin-binding profiles of MUC21 expressed by CHO-K1 cells. The left panel shows the profiles of MUC21 before and after the cell lysates were treated with sialidase (S− or S+), electrophoretically separated, and detected with a 1:1 mixture of mAbs heM21C (21C) and heM21D (21D). The middle panel indicates the profile of MUC21 immunoprecipitated with a mixture of mAbs heM21C and heM21D, electrophoretically separated, blotted, and stained with biotinylated VVA. The right panel shows the result of a similar experiment but stained with PNA instead of VVA. The arrowheads tentatively indicate the position of sialidase-treated (top) and -untreated (bottom) sialyl T-MUC21. M; mock-transfected cell clone, T; MUC21-transfected cell clone. **b** Percentage of apoptotic cells among mock-transfectant cells and two clones of CHO-K1 cells transfected with full-length MUC21 (MUC21-1, MUC21-2) after treatment with 250 μM etoposide for 48 h as determined by propidium iodide (PI) staining and binding of annexin V. Three independent experiments are shown. The mean is indicated as a horizontal line. Two-way ANOVA with Tukey’s multiple-comparison test. **c** Electrophoretic and lectin-binding profiles of MUC21 expressed by CHO-Lec2 cells. In all panels, S− and S+ show the profiles of MUC21 before (S−) and after (S+) the cell lysates were treated with sialidase. In all panels, MUC21 was immunoprecipitated with a 1:1 mixture of mAbs heM21C (21C) and heM21D (21D), electrophoretically separated, and blotted. In the left panel, the blotted membrane was stained with a 1:1 mixture of mAbs heM21C and heM21D. In the middle panel, the membrane was reacted with biotinylated VVA. In the right panel, biotinylated PNA was used. The arrowheads tentatively indicate the position of nonsialylated T-MUC21 (top) and nonglycosylated MUC21 (bottom). M; mock-transfected cell clone, T; MUC21-transfected cell clone. (**d**) Percentage of apoptotic CHO-Lec2 cells transfected with full-length MUC21 (clone MUC21-1 and MUC21-2) or mock transfectant after treatment with 250 μM etoposide for 31 h as determined by PI staining and binding of annexin V. Three independent experiments are shown. The mean is indicated as a horizontal line. Two-way ANOVA with Tukey’s multiple-comparison test. **p < 0.01, ***p < 0.001, n.s. not significant, IP immunoprecipitation, IB immunoblot, LB lectin blot, PNA peanut *(Arachis hypogaea)* agglutinin, VVA: *Vicia villosa* agglutinin.

To investigate the involvement of *O*-glycosylation in the antiapoptotic effect of MUC21, we utilized a panel of CHO-variant cells. First, we used CHO-K1 cells for the transfection of full-length MUC21 and these cells were subjected to etoposide treatment. As CHO-K1 cells can synthesize sialylated *O*-glycans, the glycosylation status of transfectant cells was first confirmed by Western blotting. Lysates from CHO-K1 full-length MUC21 were treated with or without sialidase and then immunoprecipitated with a 1:1 mixture of heM21C and heM21D. As described above, this antibody mixture recognizes nonglycosylated MUC21 and MUC21 carrying Tn, T, or sialyl T, and was used to confirm the presence of any of these specific glycoforms. Cell lysate was then immunoblotted with a 1:1 mixture of heM21C and heM21D, showing a band with a migration position corresponding to an apparent molecular weight close to 200 kDa under no sialidase-treatment condition (Fig. 5a). When cell lysate was sialidase-treated and then subjected to SDS-PAGE and immunoblotting, the band appeared at a higher apparent molecular weight, in agreement with the loss of negative charge accompanying sialic acid removal (Fig. 5a, left panel). Lectin blotting of cell lysate with *Vicia villosa* lectin (VVA) after immunoprecipitation showed no band (Fig. 5a, middle panel), but blotting with peanut (*Arachis hypogaea)* lectin (PNA) (Fig. 5a, right panel) showed a band with a migration distance corresponding to an apparent molecular weight above 200 kDa only in the sialidase-treated lysate. Together, the results suggest that sialyl T-MUC21 was expressed. Analysis of apoptotic cells after etoposide treatment of cells by flow cytometry using PI/annexin-V staining revealed that the expression of full-length sialylated MUC21 rendered the cells resistant to etoposide-induced apoptosis (Fig. 5b).

### Sialylation of *O*-glycans on MUC21 is not a prerequisite for the antiapoptotic effect

To check if sialylation of MUC21 is necessary for its antiapoptotic effect, we used Lec2 cells, CHO-derived glycosylation variant cells. Lec2 cells lack sialylation of glycans due to a reduced transport of CMP–sialic acid into the Golgi lumen [22]. Lysates from Lec2 full-length MUC21-transfected cells were treated with or without sialidase and then immunoprecipitated with a 1:1 mixture of heM21C and heM21D mAbs. Immunoprecipitated material was immunoblotted with a 1:1 mixture of heM21C and heM21D, resulting in two major bands, one at a migration distance corresponding to an apparent molecular weight around 160 kDa and one above 200 kDa, with the latter one observed regardless of sialidase treatment (Fig. 5c, left panel). Blotting with VVA did not show any bands (Fig. 5c, middle panel), while blotting with PNA resulted in a band with a migration distance corresponding to an apparent molecular weight above 200 kDa, both in nontreated and sialidase-treated lysate (Fig. 5c, right panel). Together, these results indicate that Lec2 cells expressed nonsialylated T-MUC21. Analysis of apoptotic cells after etoposide treatment of cells by flow cytometry using PI/annexin-V staining revealed that the expression of nonsialylated full-length T-MUC21 rendered the cells resistant to etoposide-induced apoptosis (Fig. 5d).

### Addition of galactose to *O*-glycans on MUC21 is necessary to elicit the antiapoptotic effect

**Fig. 6:**
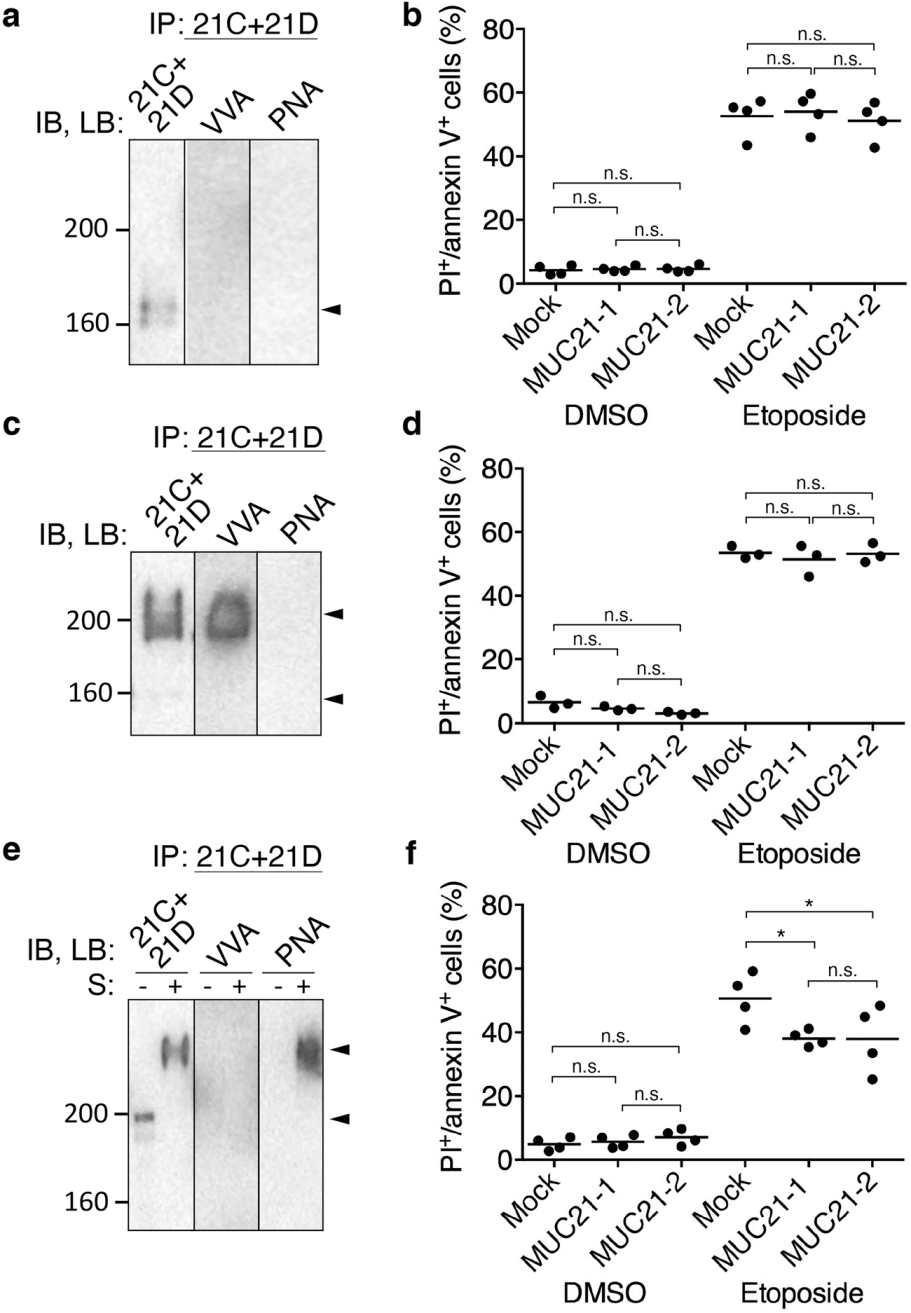
Glycosylation and antiapoptotic effects of MUC21 expressed on CHO-ldlD cells grown in different culture media. **a** Electrophoretic and lectin-binding profiles of full-length MUC21 expressed by CHO-ldlD cells grown in regular culture medium. In the middle and right panel, MUC21 was immunoprecipitated with a 1:1 mixture of mAbs heM21C (21C) and heM21D (21D), electrophoretically separated, and blotted. In the left panel, the blotted membrane was stained with a 1:1 mixture of mAbs heM21C and heM21D. In the middle and right panel, the membrane was reacted with biotinylated VVA and biotinylated PNA, respectively. The arrow head tentatively indicates the position of nonglycosylated MUC21. **b** Percentage of apoptotic CHO-ldlD cells transfected with full-length MUC21 (clones MUC21-1 and MUC21-2) or mock transfectant grown in regular culture medium after treatment with 250 μM etoposide for 48 h as determined by PI staining and binding of annexin V. Four independent experiments are shown. **c** Electrophoretic and lectin-binding profiles of full-length MUC21 expressed by CHO-ldlD cells grown in culture medium supplemented with 1 mM GalNAc. In the middle and right panel, MUC21 was immunoprecipitated with a mixture of mAbs heM21C and heM21D, electrophoretically separated, and blotted. In the left panel, the blotted membrane was stained with a 1:1 mixture of mAbs heM21C and heM21D. In the middle and right panel, the membrane was reacted with biotinylated VVA and biotinylated PNA, respectively. The arrowheads tentatively indicate the position of Tn-MUC21 (top) and nonglycosylated MUC21 (bottom). **d** Percentage of apoptotic CHO-ldlD cells transfected with full-length MUC21 or mock transfectants grown in culture medium supplemented with 1 mM GalNAc after treatment with 250 μM etoposide for 48 h as determined by PI staining and binding of annexin V. Three independent experiments are shown. **e** Electrophoretic and lectin-binding profiles of full-length MUC21 expressed by CHO-ldlD cells grown in culture medium supplemented with 1 mM GalNAc and 25 mM Gal. In all panels, cells were used untreated (S:−) or after treatment with sialidase (S:+). MUC21 was immunoprecipitated with a mixture of mAbs heM21C and heM21D, electrophoretically separated, and blotted. In the left panel, the blotted membrane was stained with a mixture of mAbs heM21C and heM21D. In the middle and right panel, the membrane was reacted with biotinylated VVA and biotinylated PNA, respectively. The arrowheads tentatively indicate the position of sialidase-treated (top) and -untreated (bottom) sialyl T-MUC21. **f** Percentage of apoptotic CHO-ldlD cells transfected with full-length MUC21 or mock transfectants grown in culture medium supplemented with 1 mM GalNAc and 25 mM Gal after treatment with 250 μM etoposide for 48 h as determined by PI staining and binding of annexin V. Four independent experiments are shown. The mean is indicated as a horizontal line. Two-way ANOVA with Tukey’s multiple-comparison test. n.s.: not significant, *p < 0.05. IP: immunoprecipitation, IB: immunoblot, LB: lectin blot, S: sialidase treatment, PNA: peanut *(Arachis hypogaea)* agglutinin, VVA: *Vicia villosa* agglutinin.

To investigate whether the extension of *O*-glycans with galactose is necessary for the apoptotic effect of MUC21, we utilized CHO-glycosylation variant ldlD cells. ldlD cells can synthesize *O*-glycans only after addition of GalNAc and/or galactose to the culture medium due to a deficiency in the enzyme UDP galactose and UDP-Gal /UDP-GalNAc 4-epimerase [23]. Full-length MUC21 was transfected into ldlD cells and cells were grown in different media to manipulate MUC21 *O*-glycosylation. Lysates of ldlD–MUC21 transfectants grown in regular medium, GalNAc-supplemented medium, and GalNAc plus Gal-supplemented medium were subjected to immunoprecipitation with a 1:1 mixture of heM21C and heM21D mAbs. The immunoprecipitated material was then blotted with a 1:1 mixture of heM21C and heM21D, VVA, and PNA to visualize the glycosylation status of MUC21. ldlD–MUC21 transfectants grown in regular medium showed only one band with a migration distance corresponding to an apparent molecular weight around 160 kDa, indicating that ldlD cells expressed only nonglycosylated MUC21 (Fig. 6a). When the medium was supplemented with GalNAc, bands with a migration distance corresponding to an apparent molecular weight around 200 kDa were seen when lysate was blotted with a 1:1 mixture of heM21C and heM21D or VVA lectin, suggesting that MUC21 carried Tn (Fig. 6c). When the medium was supplemented with GalNAc plus Gal, the 1:1 heM21C and heM21D mAb mixture recognized two bands, one with a migration distance corresponding to approximately 200 kDa (no sialidase treatment) and one above 200 kDa (after sialidase treatment). The latter band was also seen when PNA lectin was used for blotting, albeit only after sialidase treatment (Fig. 6e). These results indicate that GalNAc plus Gal supplementation led to the expression of sialyl T-MUC21. ldlD–MUC21 transfectants expressing nonglycosylated MUC21, Tn-MUC21, or sialyl T-MUC21 were subjected to etoposide treatment and analysis of apoptotic cells by flow cytometry using PI/annexin-V staining. Expression of nonglycosylated MUC21 did not render the cells resistant to etoposide-induced apoptosis (Fig. 6b). ldlD–MUC21 transfectants grown in GalNAc-supplemented medium were expressing Tn-MUC21, but these cells also did not show any resistance to etoposide-induced apoptosis (Fig. 6d). ldlD–MUC21 transfectants grown in GalNAc plus Gal-supplemented medium were expressing sialyl T-MUC21, which rendered both transfectant clones resistant to etoposide-induced apoptosis (Fig. 6f).

### Galectin-3 is not involved in MUC21-dependent induction of apoptosis resistance

**Fig. 7:**
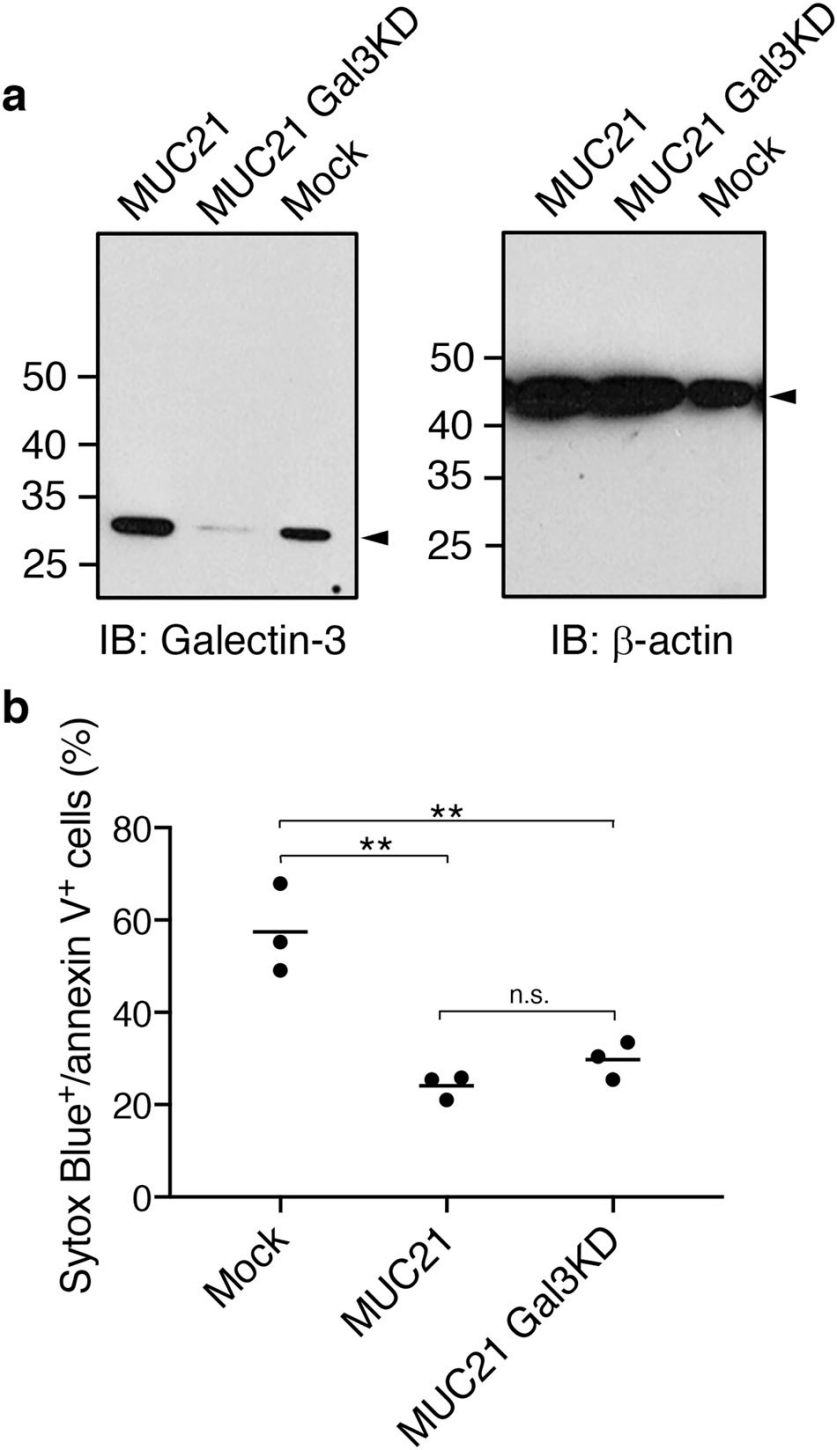
Galectin-3 silencing by shRNA does not alter the MUC21-dependent apoptosis resistance in HEK293 cells. **a** To confirm the knockdown of galectin-3 by shRNA, equal amounts of protein from cell lysates of mock transfectants (Mock), full-length MUC21 transfectants (MUC21,) and galectin-3-silenced full-length MUC21 transfectants (MUC21 Gal3KD) were separated by SDS-PAGE, blotted onto nitrocellulose membranes, and probed with anti-galectin-3 mAb TIB166 (left panel) or anti-β-actin mAb AC-74 (right panel). The arrowheads indicate the position of galectin-3 (left panel) and β-actin (right panel). **b** Mock cells, MUC21 transfectants, and MUC21Gal3KD transfectants were treated with 100 μM etoposide for 24 h. Cells were stained with Sytox Blue and annexin V, and the percentage of apoptotic cells was analyzed. Three independent experiments are shown. The mean is indicated as a horizontal line. One-way ANOVA with Tukey’s multiple-comparison test. **p < 0.01, n.s. not significant.

To investigate the mechanism by which MUC21 can induce apoptosis resistance, we speculated that galectins, which are known to be natural endogenous ligands of mucins [24, 25] and form complexes on the cell surface that cross-link glycosylated ligands and modulate their mobility [26], might be involved. Galectin-3 has been shown to have antiapoptotic effects in a variety of cell types [27–31]. In addition, interaction of circulating galectin-3 with cancer cell surface MUC1 was shown to prevent anoikis [32]. To this end, we investigated if galectin-3 is involved in the induction of MUC21-dependent apoptosis resistance by silencing galectin-3 in MUC21-transfectant HEK293 cells using shRNA. Knockdown of galectin-3 protein expression was confirmed by Western blotting (Fig. 7a). Mock-transfectant cells, MUC21-transfectant cells, and galectin-3-silenced MUC21-transfectant cells were subjected to etoposide treatment. Analysis of apoptotic cells by Sytox Blue/annexin-V staining revealed that galectin-3 silencing did not significantly alter the amount of apoptosis resistance in MUC21-transfectant cells (Fig. 7b).

## Discussion

The effects of the expression of human MUC21, a membrane-type mucin unique to squamous epithelia, on resistance of cells to apoptosis, were investigated. The present report is the first to show that MUC21 has protective effects against apoptosis, though other mucins were previously reported to confer resistance to apoptosis as elaborated below. These previous reports did not clearly show whether the glycoforms of mucins are involved in their antiapoptotic effects. Therefore, the present report is also the first to show that extension of *O*-glycans, at least galactosylation of GalNAc, is necessary in the antiapoptotic effects. Although we used etoposide to induce apoptosis in most of the experiments, the anti-cell death effect of MUC21 was also similarly observed when ultraviolet irradiation was used to induce cell death, indicating that the effect was not dependent on the transport or metabolism of this drug.

Among other mucins, involvement of MUC1 in malignant behaviors of carcinomas is widely acknowledged. This seems to have a connection to the early discovery that expression of MUC1 is associated with progression of a variety of carcinomas [33–35]. Mucins were also implicated with resistance of carcinomas to chemotherapeutic drugs, though most of the evidence was derived from experimental studies in vitro [17, 18]. Involvement of specific glycoforms of MUC1 and other *O*-glycosylated proteins has not been systematically investigated, due at least in part to the lack of tools to assess mucin glycoforms [36].

MUC1 has a cytoplasmic tail that interacts with a variety of signaling molecules [37], and it was believed that such interactions are essential in generating resistance to apoptosis. For example, MUC1 was shown to promote radioresistance in hepatocellular carcinoma cells through activation of JAK2/STAT3 signaling [38]. MUC1CT has also been proposed to localize to mitochondrial membranes under conditions of genotoxic stress, where it attenuates the apoptotic pathway and confers resistance to apoptosis-inducing drugs [39]. MUC1 activates JNK1 and inhibits apoptosis under genotoxic stress [40].

Regarding mucins other than MUC1, pancreatic cancer cells become resistant to gemcitabine in parallel with MUC4 expression [4], and MUC13 overexpression in renal cell carcinoma was reported to play a central role in tumor progression and drug resistance [6]. However, roles of MUC21 in resistance of cells to apoptosis were not previously reported. Whether the extracellular domain and its differential glycosylation is critical in determining these important phenotypes was not previously investigated.

**Fig. 8:**
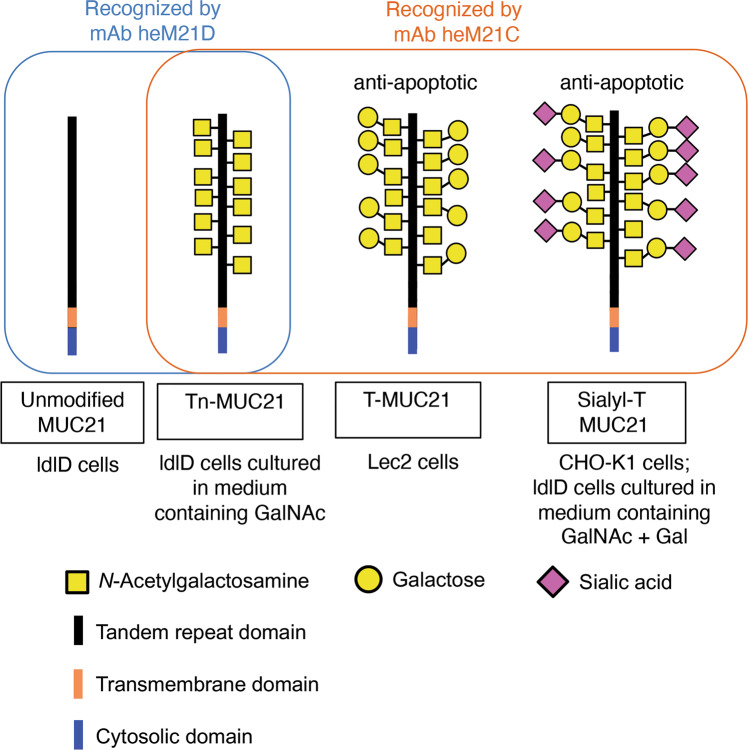
Summary of the findings of the current study. The MUC21 glycoforms expressed by the cell types and under the medium supplementation conditions used in the current study are shown together with the antiapoptotic properties of these MUC21 glycoforms. The binding specificities of monoclonal antibodies (mAb) heM21D and heM21C are indicated with a blue and a red box, respectively.

The results of our experiments by the use of truncated MUC21-expressing cells revealed that both the cytosolic and the tandem-repeat portion of MUC21 are necessary for eliciting resistance to apoptosis. Further experiments using glycosylation mutants of CHO cells revealed that the extended carbohydrate chains with Gal residues on the *O*-glycans of MUC21 are important in the antiapoptotic effects. Although culture condition-dependent changes in the structures of *O*-glycans are rather ubiquitous in that they affect all glycoproteins in a given cell type, the resistance to apoptosis was only observed when MUC21 was expressed. Therefore, it is reasonable to speculate that MUC21 with a particular glycoform is necessary to elicit such effects. As illustrated in Figs. 5 and 6 we were able to show that the glycoforms of MUC21 were completely changed, depending on whether they were expressed by CHO-K1, Lec2, or ldlD cells cultured with GalNAc, with GalNAc plus Gal, or without supplementation. Our Western blot results showed that CHO-K1 cells expressing full-length MUC21 cells and also ldlD cells expressing full-length MUC21 that were supplemented with GalNAc plus Gal expressed sialyl T-MUC21. In the case of ldlD cells supplemented with GalNAc plus Gal, both transfectant clones showed significant resistance to apoptosis (Fig. 6 f). Similarly, both CHO-K1-transfectant clones showed resistance to apoptosis (Fig. 5b), supporting the conclusion that sialyl T-MUC21 has antiapoptotic properties. Our results strongly suggest that the expression of T-MUC21 and sialyl T-MUC21 confers antiapoptotic properties to CHO-variant cells. In contrast, the expression of nonglycosylated MUC21 or Tn-MUC21 does not confer antiapoptotic properties to these cells (Fig. 8). As for MUC1, it was reported that knockdown of the C1GT enzyme, which catalyzes the addition of galactose to GalNAc attached to Thr and Ser residues, resulted in a relative decrease in T-glycans and a relative increase in Tn-glycans on the cell surface of MUC1-transfected epithelial cancer cells and resulted in increased anoikis [20]. However, the authors of that study did not determine if or to which extent particular glycoforms of MUC1 were responsible for this phenomenon. Our present study suggests that MUC21 glycoforms differ in their capability to induce resistance to apoptosis. Together with our findings that both the tandem repeat and the cytosolic tail of MUC21 are necessary to confer resistance to apoptosis, and that apoptosis resistance was also observed after exposure to UV light, our results suggest that the underlying mechanism might be based on signal transduction by MUC21 with unique glycoforms.

It remains a difficult task to elucidate the mechanism by which MUC21 can make cells resistant to apoptosis. However, the obvious requirement for extended glycans to have terminal Gal residues or to be capped with sialic acid residues in order to elicit the antiapoptotic signal suggests that the signal is potentially generated by an interaction of MUC21 with carbohydrate-recognition molecules. Therefore, we speculated that galectins or siglecs could be involved. As an initial step to gain mechanistic insight, we tested the involvement of galectin-3. We showed that silencing galectin-3 in MUC21-expressing HEK293 cells did not alter MUC21-dependent apoptosis resistance. In the case of MUC21-expressing Lec2 cells, we showed that these cells exhibited MUC21-dependent apoptosis resistance, despite the lack of sialic acid, suggesting that siglecs might not be necessary for the apoptosis resistance to occur, at least in Lec2 cells. Public data in the Human Protein Atlas show that HEK293 cells express low amounts of siglec 10 and 11. Siglec 10 was shown to specifically bind α2,3- or α2,6-linked sialic acid [41], while siglec 11 specifically recognizes α2,8-linked sialic acid [42]. On the other hand, CHO cells were shown to synthesize relatively large amounts of galectin-1 [43] and to quantitatively externalize it on the cell surface where it can bind glycoconjugates [44]. In this respect, our galectin-3 findings provide an important yet limited first step in elucidating the mechanistic underpinning of MUC21-dependent apoptosis resistance. In the future, it remains to be determined if siglecs or other galectin-family members might mediate MUC21-dependent apoptosis resistance, depending on the cell type.

MUC21 mRNA and protein are highly expressed by esophagus, vagina, cervix, and skin as shown in the Human Protein Atlas. The mucosal surfaces in these organs are exposed to external insults, and it seems reasonable that mechanisms exist, which make surface-facing cells more resilient. Our previous report showed that *O*-glycans of MUC21 are extended during the differentiation of esophageal epithelial cells and their migration toward the lumen [21]. Thus, it is likely that these cells acquire the capacity to be sturdy during their differentiation.

In conclusion, we have shown that MUC21 expressed in CHO cells confers resistance to apoptosis in an *O*-glycosylation-dependent manner. Our findings underscore the structure–function relationship of specific mucin glycoforms. Whether MUC21 expressed by carcinoma cells is involved in malignant behavior and resistance to therapy remains to be elucidated in the clinical context.

## Materials and methods

### Cells and cell cultures

HEK293–MUC21 transfectants, HEK293-Δ-TR-MUC21 transfectants, HEK293-Δ-CT-MUC21 transfectants, and mock transfectants, obtained as described below, were grown in DMEM–high glucose with 10% FCS in a humidified atmosphere with 5% CO_2_. CHO-K1 cells and CHO-Lec2 cells, obtained from the American Tissue Culture Collection, CHO-ldlD cells, kindly provided by Dr. Monty Krieger of Massachusetts Institute of Technology, USA, and MUC21 transfectants from these cells prepared as described below were grown in a 1:1 mixture of DMEM and Ham’s F12 with 10% FCS in a humidified atmosphere with 5% CO_2_. Cell lines were routinely checked for mycoplasma contamination. Only mycoplasma-free cell lines were used in the experiments.

### Preparation of a vector construct containing full-length MUC21 and deletion variants of MUC21

A pcDNA3.1(−) vector containing the full-length human MUC21 cDNA FLAG-tagged at its amino terminal was generated as previously described [11]. To prepare vector constructs containing MUC21 with a deleted tandem-repeat domain (Δ-TR) or MUC21 with a deleted cytoplasmic domain (Δ-CT), specific forward primers and reverse primers were used to amplify the truncated region. The primers used for 3 x FLAG-MUC21-Δ-TR were MUC21-Δ-TR-Forward; 5′CGCCGTCTCTTCTGCAGGCTCTGGAACA3′; MUC21-Δ-TR-Reverse; 5′CGCCGTCTCGCAGAAGAGGTGCTAGTCTCATT3′. The primers used for 3 x FLAG-MUC21-Δ-CT were MUC21-Δ-CT-Forward; 5′CGCCGTCTCATGAGAATTCTGCAGATAT3′; MUC21-Δ-CT-Reverse; 5′CGCCGTCTCTCTCATCTCACACAGAAGAAGAG3′. After agarose gel electrophoresis (1%) in the presence of ethidium bromide, a band at 5 kb was excised and the amplified DNA was retrieved by QIAquick gel extraction kit (Qiagen, Germantown, MD). The obtained PCR products were treated with Bsm BI (New England Biolabs, Ipswich, MA) and 5-kB bands were separated by electrophoresis under the same conditions as above, and retrieved by the QIAquick gel extraction kit (Qiagen). The retrieved DNA was ligated into pcDNA^TM^3.1(+) vector with Ligation High (Toyobo, Osaka, Japan), transformed into DH5α, and subcloned. The transformed DH5α with 3 x FLAG-MUC21-pcDNA^TM^3.1(+) plasmid was propagated in LB medium. The plasmid was purified by QIAprep Miniprep (Qiagen). The sequences of 3 x FLAG-MUC21-Δ-TR and 3 x FLAG-MUC21-Δ-CT were confirmed on an ABI PRISM^R^3100 Genetic Analyzer.

### Transfection of full-length MUC21, Δ-TR-MUC21, and Δ-CT-MUC21 constructs into HEK293 cells

Transfection was performed with Lipofectamine LTX reagent (Thermo Fisher Scientific, Waltham, MA) according to the manufacturer’s instructions. The DNA–lipid complex was added to HEK293 cells previously grown overnight and incubated at 37 °C for 6 h. After this period, the cells were further incubated with medium without antibiotics for 18 h, passaged, incubated for 24 h, and then placed into media containing G418 (800 µg/ml). G418 selection was conducted for one week, and the resultant cells were cloned by the limiting-dilution method.

### Preparation of full-length MUC21, Δ-CT-MUC21, and Δ-TR-MUC21 transfectants from CHO-K1, CHO-Lec2, and CHO-ldlD cells

Transfection of full-length MUC21, Δ-CT-MUC21, and Δ-TR-MUC21 into CHO-K1, CHO-Lec2, and CHO-ldlD cells, selection, and cloning were performed as previously reported [21]. In the case of CHO-K1 and CHO-Lec2 cells, both nontagged and N-FLAG-tagged MUC21-transfectant clones were used in the experiments described below.

### Flow cytometric analysis

FACS buffer was prepared by dissolving 0.1% BSA in PBS. Binding buffer for Annexin-V/PI staining contained 10 mM HEPES, 140 mM NaCl, and 2.5 mM CaCl_2_, and was adjusted to pH 7.2. Detached cells were rinsed with FACS buffer and blocked with FACS buffer containing 10% normal goat serum (Japan Laboratory Animals, Inc., Tokyo, Japan) at 4 °C for 10 min. Anti-FLAG mAb (clone M2, F1804, Sigma, St. Louis, MO), purified mouse IgG1 (Thermo Fisher Scientific, Waltham, MA), or a 1:1 mixture of supernatants of hybridomas heM21C and heM21D, was added and incubated at room temperature for 30 min. After washing with FACS buffer, the cells were incubated with FITC goat anti-mouse IgG (H + L) (Invitrogen) diluted in FACS buffer containing 5% normal goat serum and incubated at 4 °C for 30 min. The cells were washed with FACS buffer, passed through a nylon mesh, and analyzed with an Epics XL (Beckmann Coulter, Brea, CA).

### Sialidase treatment of cell suspensions

Cells were recovered, washed twice with PBS, and suspended in phosphate buffer (pH 5.8). Sialidase (derived from *Clostridium perfringens*, Sigma) was added at a concentration of 100 mU/ml and reacted at 37 °C for 30 min.

### Preparation of cell lysates

For experiments shown in Figs. 2, 3, 5, and 6, desalting buffer (0.25 M sucrose, 10 mM Tris-HCl, and 0.05 mM CaCl_2_, pH 7.2) was prepared. Detached cells were rinsed with PBS and lysed in 0.5% NP-40 (Sigma) in desalting buffer containing protease-inhibitor cocktail set III (Calbiochem, San Diego, CA) (1:100). The sample was rotated overnight at 4 °C, and then the supernatants were obtained by centrifugation. Protein contents of the lysates were determined by the BCA protein- quantification reagent (Pierce, Waltham, MA). For experiments shown in Fig. 7, protein lysates were prepared using CytoBuster protein-extraction buffer (Millipore, Burlington, MA) according to the manufacturer’s instructions.

### Western and lectin-blotting analysis

To elucidate which MUC21 glycoforms are expressed in the cell lines and after the enzyme treatments used, we employed immunoprecipitation of MUC21 protein followed by a combined Western blotting and lectin-blotting analysis of the precipitated material. For experiments shown in Figs. 2, 3, 5, and 6, SDS-PAGE was performed on 6% polyacrylamide gels under reducing conditions. The separated materials were blotted onto PVDF membranes (ImmobilonTM, Millipore) under semidry conditions. After blocking with 3% BSA at 4 °C overnight, the membranes were incubated with anti-FLAG M2 mAb (F1804, Sigma) (1:1000), or hybridoma culture supernatants heM21C and heM21D. Antibodies were diluted in 3% BSA in 0.1% Tween20–PBS. After washing with 0.1% Tween20–PBS for 30 min, the membranes were incubated with HRP-goat anti-mouse IgG (H + L) (Thermo Fisher Scientific) or HRP-goat anti-rabbit IgG (H + L) (Thermo Fisher Scientific) diluted (1:1000) in 3% BSA in 0.1% Tween20–PBS at room temperature for 45 min. After washing for at least 2 h, HRP coloring reagents and Amersham Hyperfilm ECL (GE Healthcare, Chicago, IL) were used to visualize antibody reactivity. Lectin-blotting analysis was performed with biotinylated *Vicia villosa* agglutinin (VVA) (Vector Laboratories, Burlingame, CA) and with peanut (*Arachis hypogaea)* agglutinin (PNA) (J-OIL MILLS, Tokyo, Japan). In some cases, cell lysates were treated with sialidase (purified from *Clostridium perfringens*). For experiments shown in Fig. 7, SDS-PAGE was performed on 10% polyacrylamide gels (Thermo Fisher Scientific) under reducing conditions. The separated materials were blotted onto iBlot 2 NC mini stacks using the iBlot 2 transfer apparatus (Thermo Scientific, Waltham, MA). After blocking with protein-free T20 blocking buffer (Thermo Scientific) for 1 h at room temperature, the membranes were washed three times for 5 min with TBST and then incubated with anti-galectin-3 mAb (clone M3/38.1.2.8 HL.2, ATCC® TIB166™), purified from culture medium of TIB166 rat hybridoma cells, and obtained from the American Type Culture Collection (1:500), or anti-human beta-actin mAb (clone AC-74, A5316, Sigma) (1:1000) diluted in TBST for 1 h. After washing three times for 10 min with TBST, the membranes were incubated with HRP-goat anti-rat IgG or HRP-rabbit anti-mouse IgG diluted (1:2000) in TBST at room temperature for 1 h. After washing with TBST three times for 15 min, blots were rinsed once with TBS and then Amersham Hyperfilm ECL was used to visualize antibody reactivity. Blots were exposed to BioMax light film (Carestream, Rochester, NY).

### Induction of apoptosis by chemotherapeutic agents

Etoposide (Sigma) was used to induce apoptosis. For experiments shown in Figs. 1–3, 5 and 6, cells transfected with full-length MUC21, Δ-TR-MUC21, or Δ-CT-MUC21, and mock transfectants were seeded into six-well plates at 3 × 10^5^ cells/well and incubated with etoposide at a final concentration of 100 µM. Both attached and floating cells were harvested. The cells were washed once with binding buffer. Then 100 µl of Annexin V–Biotin (MBL, Nagoya, Japan) (1:50) was added and cells were incubated at room temperature for 15 min. After rinsing with binding buffer, 100 µl of FITC–streptavidin (Invitrogen) (1:200) was added. After 30 min of incubation at room temperature, cells were washed once and 100 µl of 1 µg/ml propidium iodide solution was added. The cells were passed through a nylon mesh and analyzed with an Epics XL (Beckman Coulter). For experiments shown in Fig. 7, Annexin V–FITC (BD Bioscience, San Jose, CA) and Sytox Blue (Thermo Fisher Scientific) were used instead, and the samples were analyzed with a BD FACSCelesta (BD Biosciences).

### Induction of cell death by UV irradiation

The cells were seeded at 3 × 10^5^ cells/well on six-well plates and incubated overnight. After the culture supernatants were removed, the cells were rinsed once with PBS and placed under sterilizing UVC light (Hitachi GL-15, emitting a wavelength of 253.7 nm) (Hitachi, Tokyo, Japan) at a distance of 5 cm. Culture medium was added and cells were cultured for 24 h, after which dead cells were assessed by flow cytometry with PI staining. The intensity of UV was monitored by a Photodiode Sensor (OPHIR, PD300-UV). At the distance of 5 cm, the intensity was 17.23 nW/cm^2^. In these experiments, the radiation exposure was set to be 2.6, 6.0, and 10.3 J/m^2^.

### Silencing of galectin-3 in cloned MUC21-transfeced HEK293 cells by shRNA

Silencing of galectin-3 was done using lentivirus packaging for shRNA against human NM_022306.2, i.e., LPP-HSH010590-LVRH1MP-100-m and scrambled control lentiviral particles for psi-LVRH1MP LPP-CSHCTR0001-LVRH1MP-100-C (GeneCopoiea, Rockville, MD). On Day 1, 5 × 10^4^ cells in 3 ml of Opti-MEM medium were seeded per well in a six-well dish. On Day 2, 5 μl of lentivirus were mixed with 2 μl of 4 μg/ml polybrene and 994 μl of Opti-MEM medium and incubated for 5 min at room temperature. Two ml of this mixture was added to each well. On Day 3, 2 ml of fresh Opti-MEM medium was replaced in each well. On Day 5, culture medium was replaced with 2 ml of 10 μg/ml puromycin in fresh Opti-MEM medium. Puromycin-containing medium was replaced daily for three weeks. The silenced cells were used without cloning.

### Statistical analysis

Statistical analysis was performed using two-way ANOVA followed by Tukey’s multiple-comparison test for experiments analyzing apoptosis resistance in MUC21-transfected cells involving DMSO and etoposide as treatment groups. Ordinary one-way ANOVA was used to analyze differences in apoptosis resistance in MUC21-transfected cells after shRNA knockdown. Shapiro–Wilk test was used to test if the data are normally distributed. Brown–Forsythe test and/or Bartlett’s test were used to test if the standard deviations of the data, which were statistically compared, were similar. All data were analyzed using PRISM 8 software. The significance level was set to *p* < 0.05.

## Supplementary information

Original data

## Data Availability

All data generated or analyzed during this study are included in this published article.
