# Supplementary material for: Mucin 21 confers resistance to apoptosis in an O-glycosylation-dependent manner
Source: Cell Death Discov. 2022 Apr 11;8:194. doi: 10.1038/s41420-022-01006-4 (PMC9001685; doi:10.1038/s41420-022-01006-4)

Supplementary material for Tian et al.

Mucin 21 confers resistance to apoptosis in an *O*-glycosylation dependent manner

original uncropped images

Fig. 2 c

Figure as in the paper

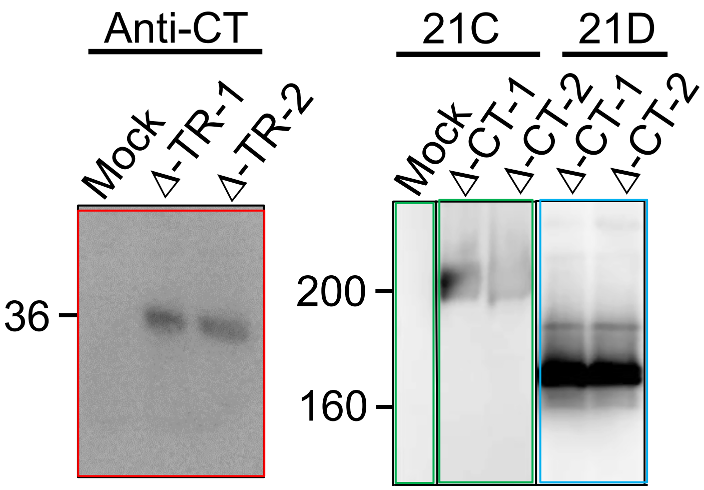

Raw image

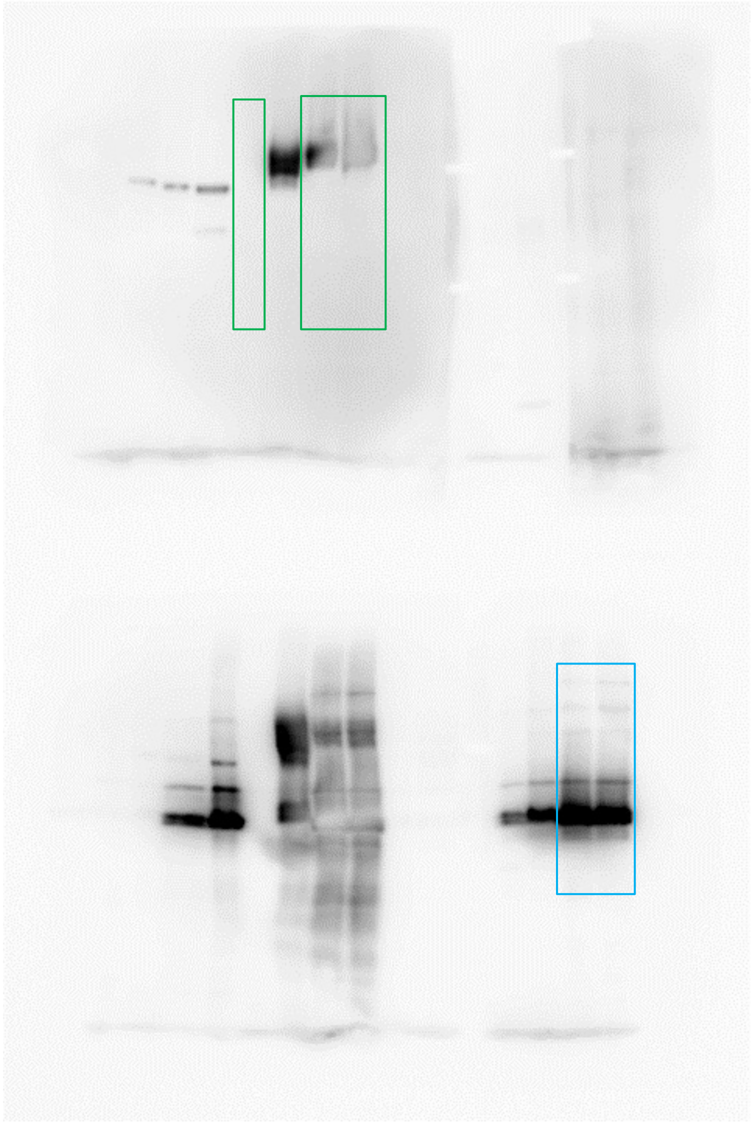

Raw image

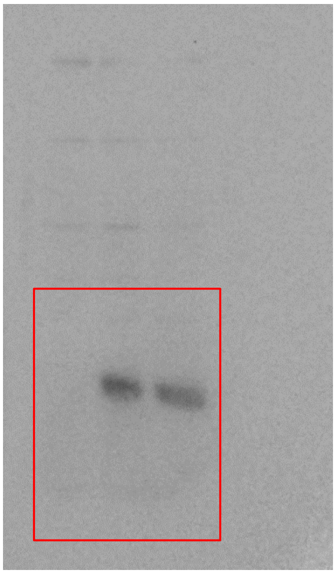

Fig. 3 a

Figure as in the paper

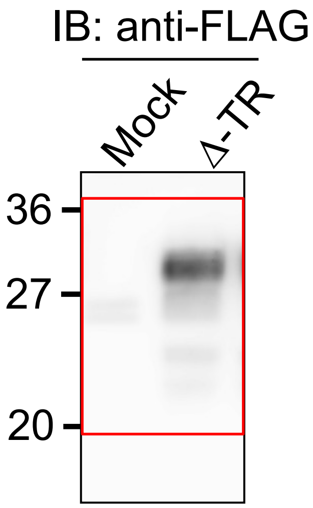

Raw image

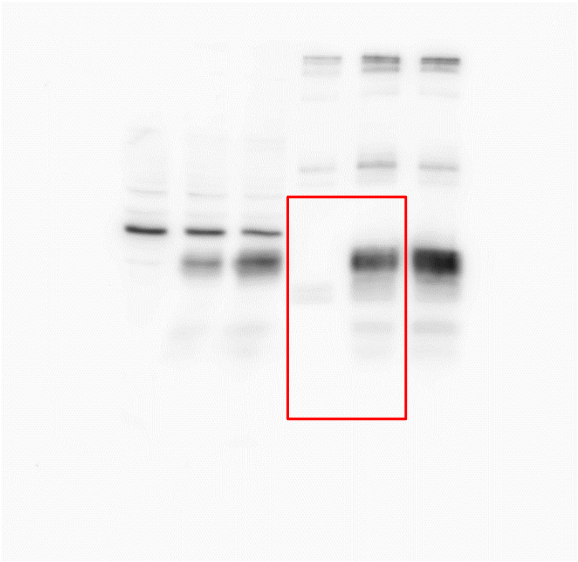

Fig. 3 b

Figure as in the paper

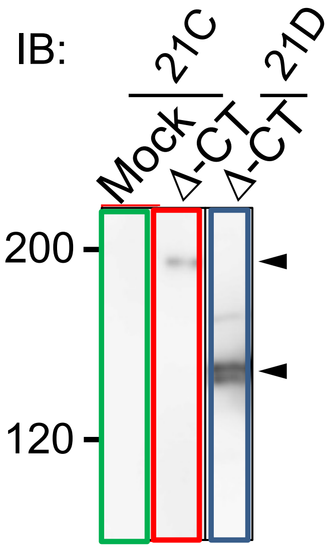

Raw image

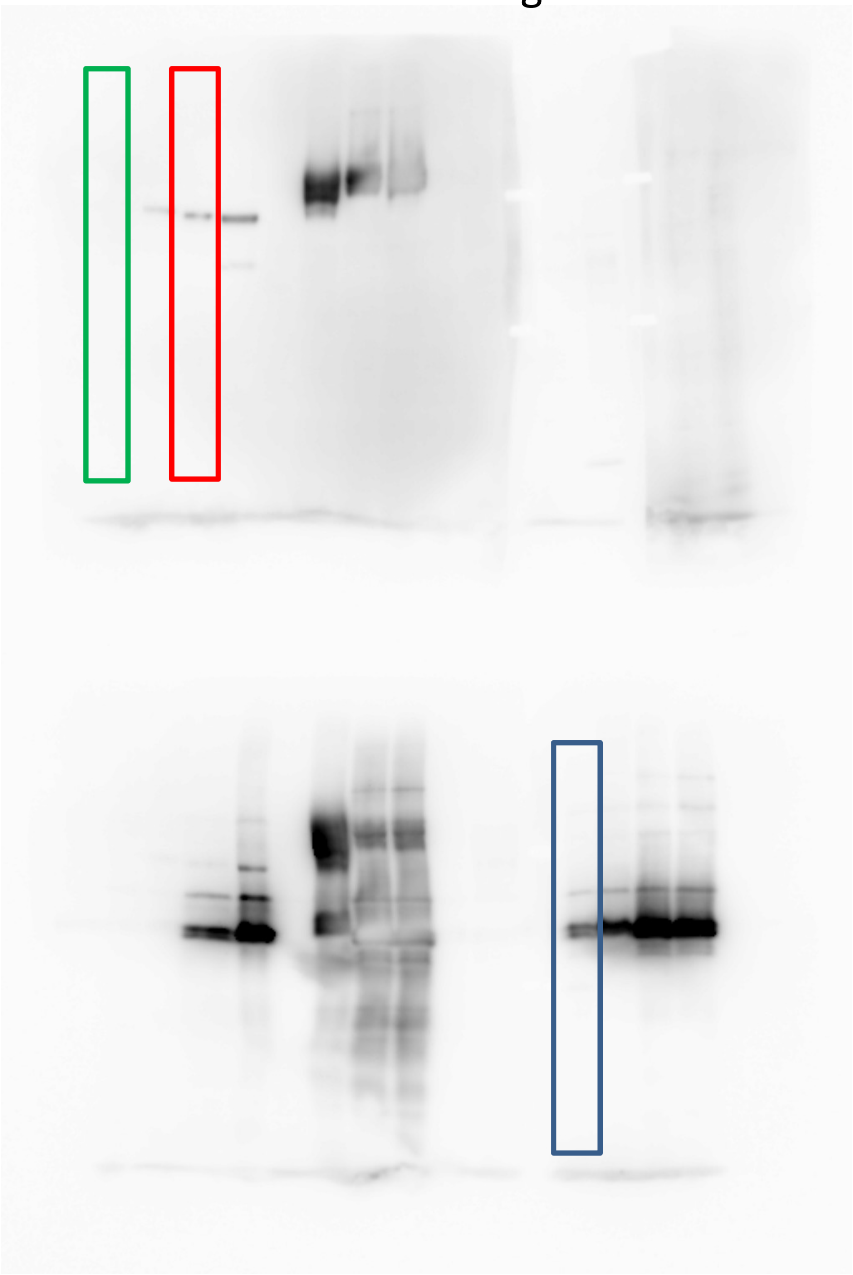

Fig. 5 a

Figure as in the paper

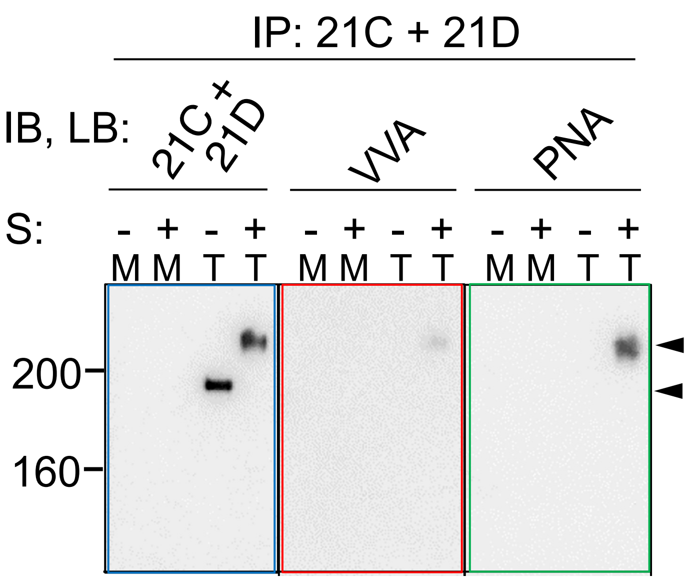

Raw image

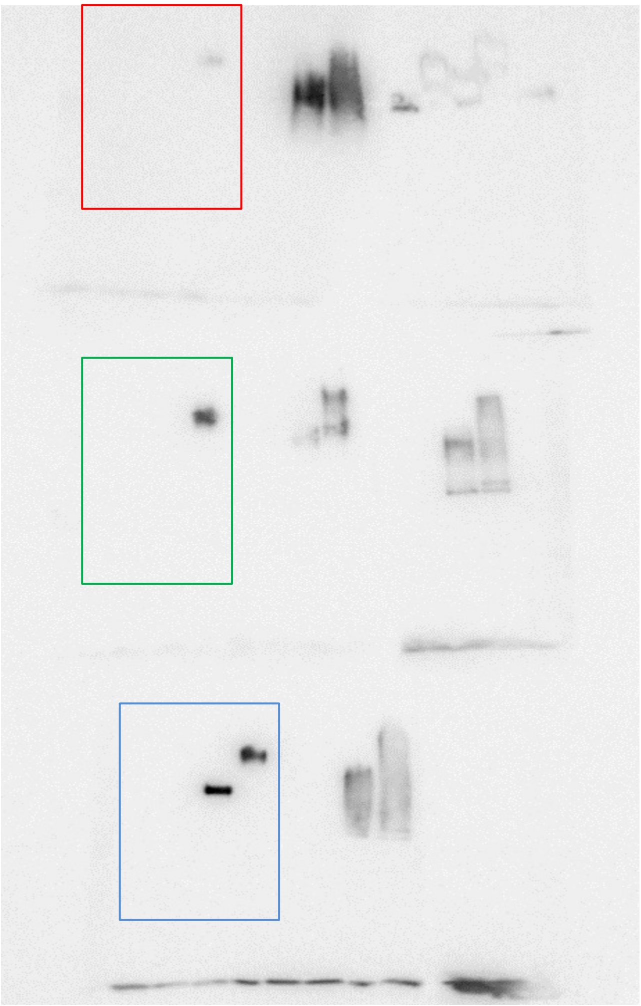

Fig. 5 c

Figure as in the paper

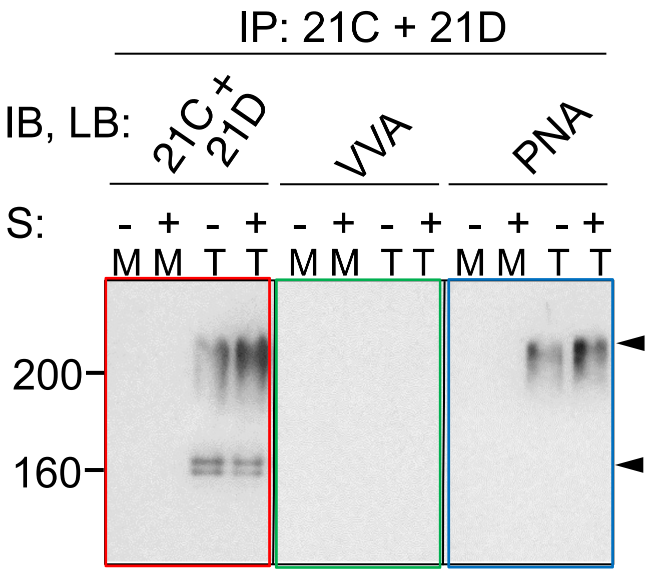

Raw image

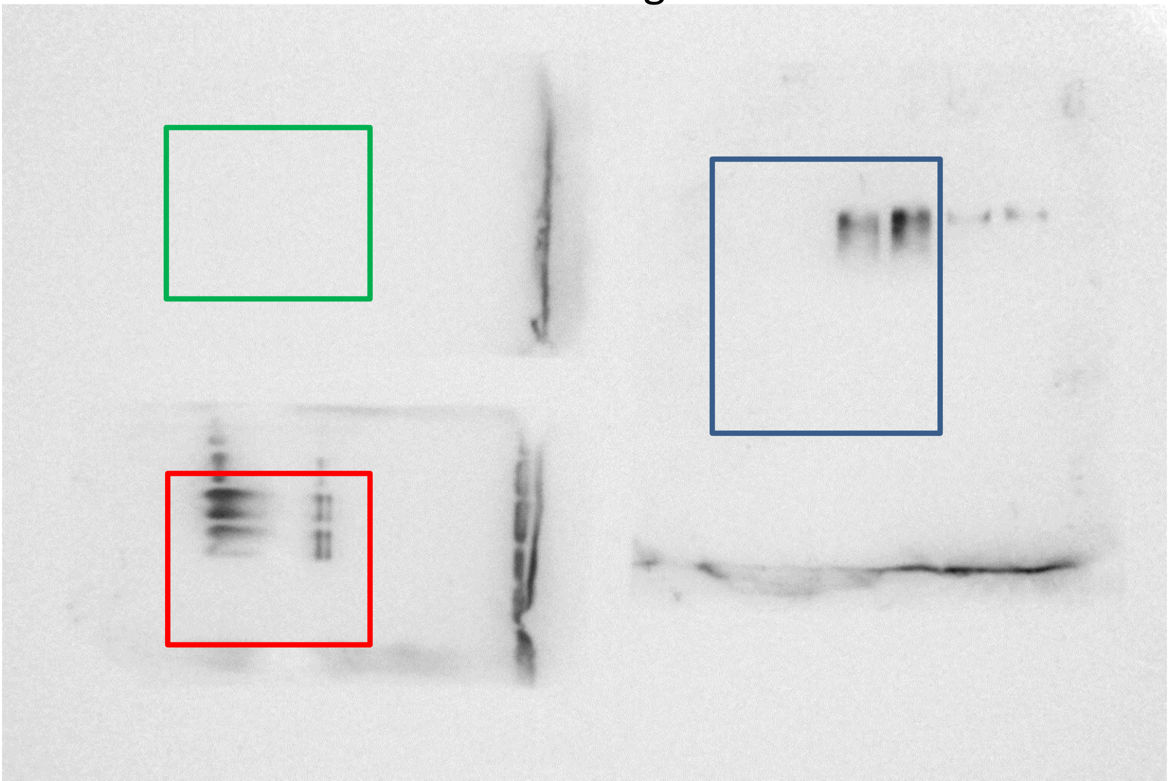

Figures as in the paper

Fig. 6 a

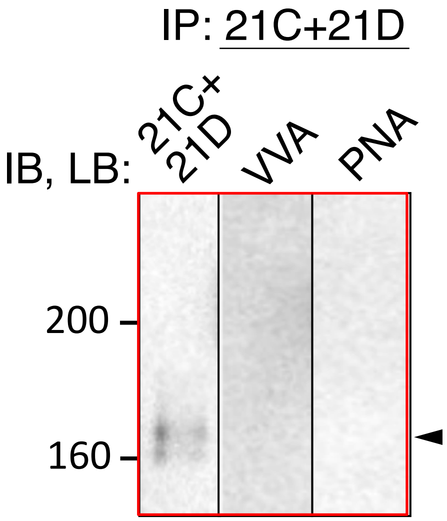

Fig. 6 c

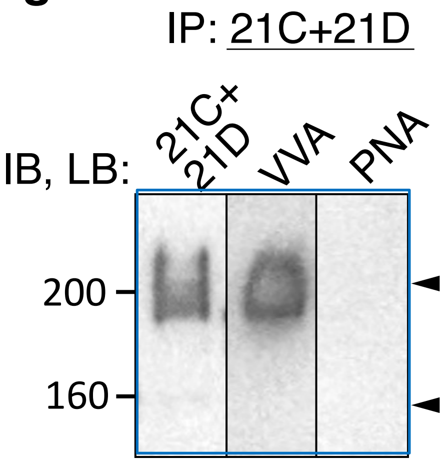

Fig. 6 e

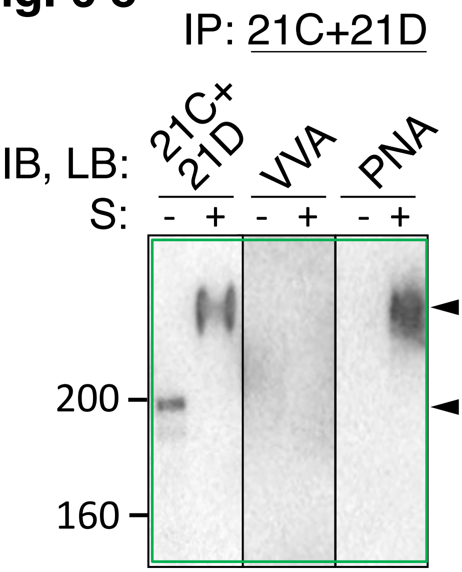

Raw image

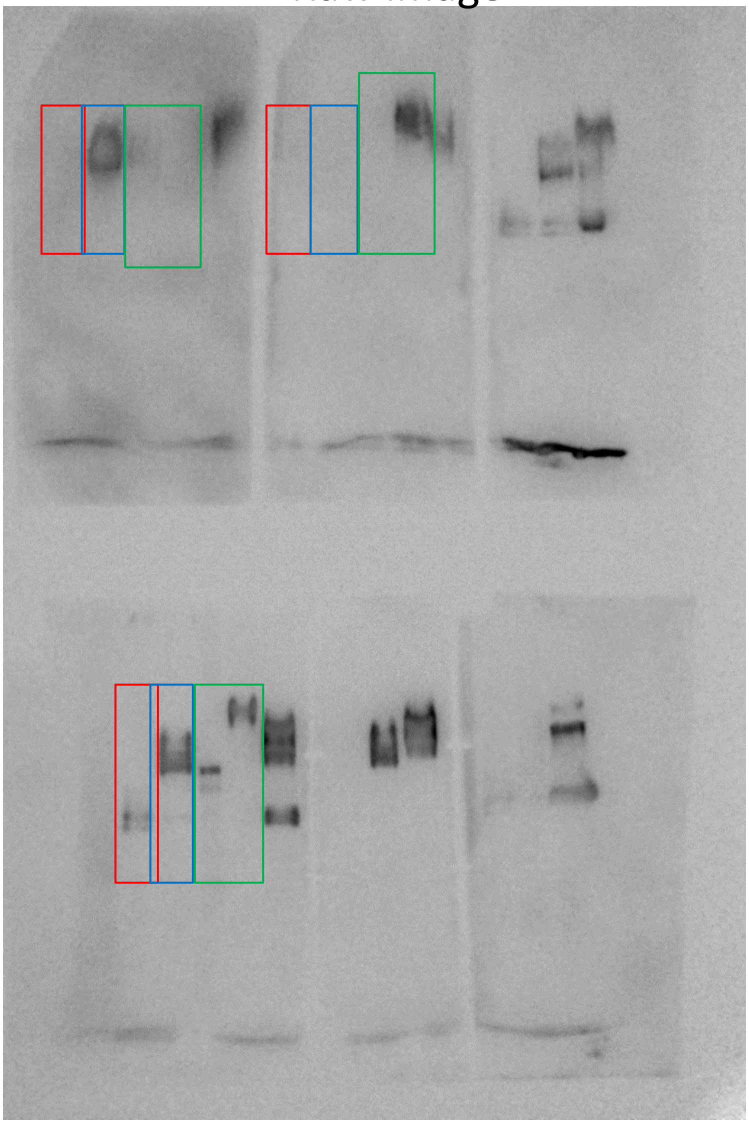

Fig. 7 a

Figure as in the paper

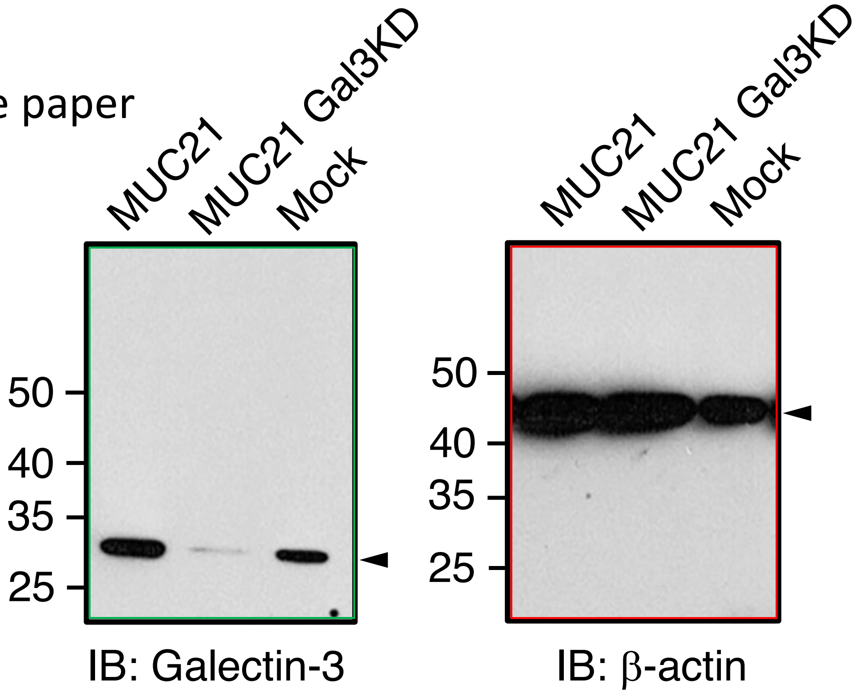

Raw image, unmanipulated

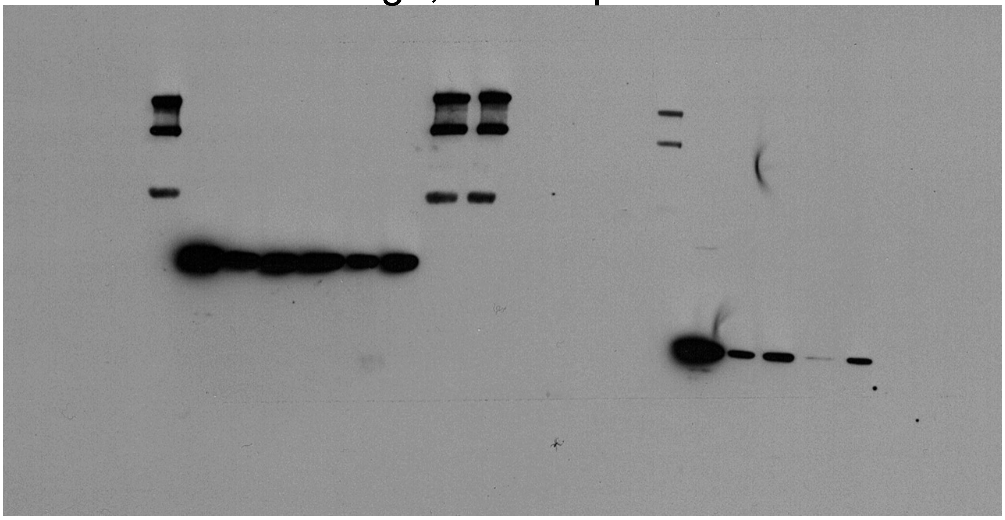

Image, manipulated by enhancing brightness and reducing contrast (both across the entire image)

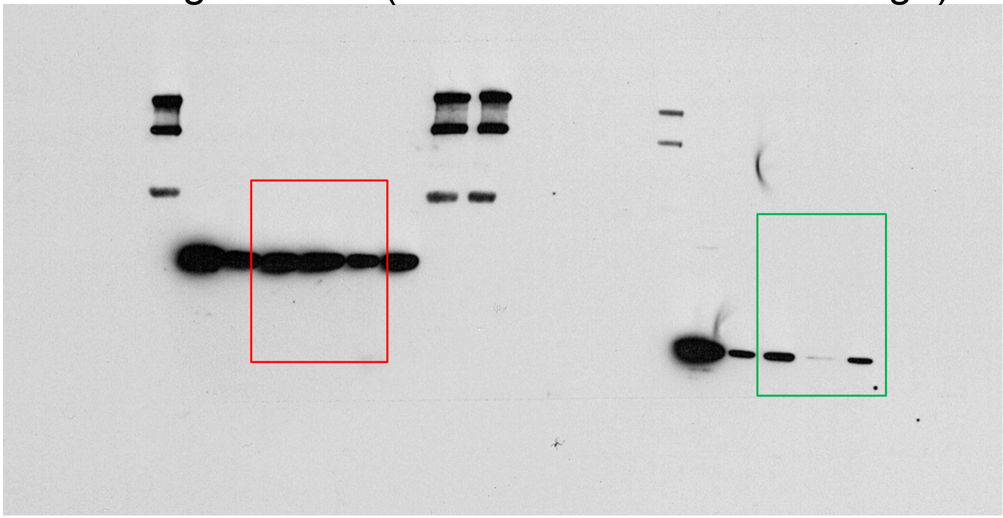

Supplement: Supplementary file 1 — Original data [file 41420_2022_1006_MOESM1_ESM.pdf]
